# A MATLAB toolbox to fit and forecast growth trajectories using phenomenological growth models: Application to epidemic outbreaks

**DOI:** 10.21203/rs.3.rs-2724940/v1

**Published:** 2023-03-27

**Authors:** Gerardo Chowell, Amanda Bleichrodt, Sushma Dahal, Amna Tariq, Kimberlyn Roosa, James M. Hyman, Ruiyan Luo

**Affiliations:** 1 Department of Population Health Sciences, School of Public Health, Georgia State University, Atlanta, GA, USA.; 2 Stanford University, School of Medicine, CA, USA; 3 National Institute for Mathematical and Biological Synthesis (NIMBioS), University of Tennessee, Knoxville, TN, USA; 4 Department of Mathematics, Center for Computational Science, Tulane University, New Orleans, LA, USA.

**Keywords:** MATLAB toolbox, real-time forecasting, dynamic growth models, GGM, GLM, Richards model, epidemic outbreak

## Abstract

**Background:**

Simple dynamic modeling tools can be useful for generating real-time short-term forecasts with quantified uncertainty of the trajectory of diverse growth processes unfolding in nature and society, including disease outbreaks.

**Results:**

In this tutorial-based primer, we introduce and illustrate a user-friendly MATLAB toolbox for fitting and forecasting time-series trajectories using phenomenological dynamic growth models based on ordinary differential equations. This toolbox is accessible to various audiences, including students training in time-series forecasting, dynamic growth modeling, parameter estimation, parameter uncertainty and identifiability, model comparison, performance metrics, and forecast evaluation, as well as researchers and policymakers who need to conduct short-term forecasts in real-time. The models included in the toolbox capture exponential and sub-exponential growth patterns that typically follow a rising pattern followed by a decline phase, a common feature of contagion processes. Models include the 2-parameter generalized-growth model, which has proved useful to characterize and forecast the ascending phase of epidemic outbreaks, as well as the 3-parameter generalized logistic-growth model and the Richards model, which have demonstrated competitive performance in forecasting single peak outbreaks.

The toolbox provides a tutorial for forecasting time-series trajectories that include the full uncertainty distribution, derived through parametric bootstrapping, which is needed to construct prediction intervals and evaluate their accuracy. Functions are available to assess forecasting performance across different models, estimation methods, error structures in the data, and forecasting horizons. The toolbox also includes functions to quantify forecasting performance using metrics that evaluate point and distributional forecasts, including the weighted interval score.

**Conclusions:**

As a contagion process takes off, the tools in the presented toolbox can facilitate policymaking to guide the implementation of control strategies and assess the impact of interventions. The toolbox functionality is demonstrated through various examples, including a tutorial video, and is illustrated using weekly data on the monkeypox epidemic in the USA.

## Background

There is a need for easy-to-use and flexible dynamic modeling tools to generate short-term forecasts with quantified uncertainty of the trajectory of diverse growth processes observed in nature and society, such as infectious disease outbreaks [1]. This tutorial paper introduces a user-friendly MATLAB toolbox to fit and forecast time-series trajectories using phenomenological dynamic growth models based on ordinary differential equations with applications in the natural and social sciences. This toolbox is written for various audiences, including students training in time-series forecasting, dynamic growth modeling, parameter estimation, parameter uncertainty and identifiability, model comparison, performance metrics, and forecast evaluation. The toolbox is also useful for researchers and policymakers who need to conduct short-term forecasts by relying on historical and real-time trajectory data of the process of interest such as an unfolding epidemic.

Unlike popular statistical time series models such as Auto Regressive Integrated Moving Average (ARIMA), this paper deals with forecasting signals using simple dynamic models based on ordinary differential equations. These models capture exponential and sub-exponential growth patterns that typically follow a rising pattern followed by a decline phase, a common feature of contagion processes [2–5]. The toolbox provides functions for forecasting trajectories with their full uncertainty distribution which is needed to construct prediction intervals and evaluate their accuracy. The toolbox includes functions that allow the user to assess forecasting performance for different models, estimation methods, error structures in the data, and forecasting horizons.

Models in the toolbox include the 2-parameter generalized-growth model [6, 7], which has proved useful to characterize and forecast the ascending phase of epidemic outbreaks [8], as well as the 3-parameter generalized logistic-growth model and the Richards model, which have demonstrated competitive performance in forecasting single peak epidemics [5, 8, 9]. The toolbox includes nonlinear least squares estimation and maximum likelihood estimation methods with different assumptions about the error structure of the observed data, including Poisson, negative binomial, and normal distributions, as well as uncertainty quantification based on a parametric bootstrapping approach [1, 9]. The toolbox also includes functions to quantify forecasting performance using metrics that evaluate point and distributional forecasts, including the weighted interval score. The toolbox’s functions are illustrated using weekly case data from the monkeypox epidemic in the USA.

This tutorial-based primer is organized as follows. First, the format of the input time-series data is described, followed by the parameter estimation methods included. Second, the underlying methodology, user parameters, and functions to calibrate, evaluate, and display model fits to data are described, including options to assess the temporal stability of model parameters through rolling window analyses. Finally, the functions to generate, display, and quantify the performance of model-based forecasts are introduced with specific examples in the context of the monkeypox epidemic in the USA. A tutorial video that demonstrates the toolbox functionality is included in the supplement (supplementary file 1).

## Implementation

### Installing the toolbox

- Download the MATLAB code located in folder ‘**forecasting_growthmodels code**’ from the GitHub repository: https://github.com/gchowell/forecasting_growthmodels
- Create ‘input’ folder in your working directory where your input data will be stored.
- Create ‘output’ folder in your working directory where the output files will be stored.
- Open a MATLAB session.

### Overview of the toolbox functions

Table 1 lists the names of both user and internal functions associated with the toolbox along with a brief description of their role.

### The input dataset

For the purposes of this toolbox, the time-series data will be stored in the ‘input’ folder and needs to be a text file with the extension *.txt. The first column should correspond to the time index: 1, 2, 3, …, and the second corresponds to the temporal incidence data. If the time series file contains cumulative incidence count data, the name of the time series data file must start with “cumulative”.

To illustrate the methodology presented in this tutorial paper, we employ the weekly incidence curve of monkeypox cases reported in the USA from the publicly available data published by the Centers for Disease Control and Prevention (CDC) from the week of 12 May 2022 through the week of 15 December 2022[10]. The data file is in the input folder within the working directory (data file path: ./input/ Most_Recent_Timeseries_US-CDC.txt). A snapshot in Excel of the contents of the file is shown below:


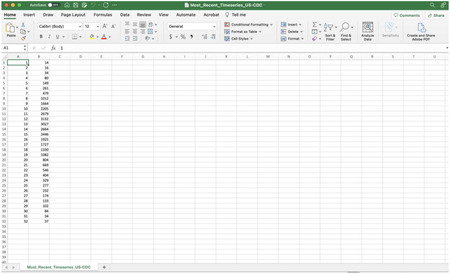


In the options_fit.m and options_forecast.m files, the user specifies the parameters related to model fitting and forecasting, respectively, as shown below. For instance, parameter <cadfilename1> is a string used to indicate the name of the data file, parameter <caddisease>, is a string used to indicate the name of the disease related to the time series data, whereas <datatype> is a string parameter indicating the nature of the data (e.g., cases, deaths, hospitalizations).


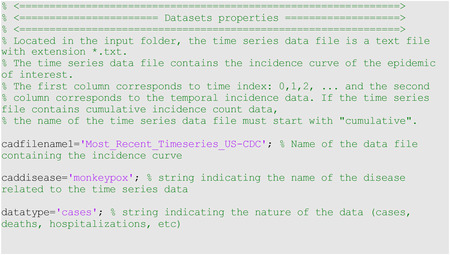


### Parameter estimation method

Let $y_{t_{j}}=y_{t_{1}},y_{t_{2}},\ldots ,y_{t_{n_{d}}}$ where *j* = 1, 2, …, *n*_*d*_ denote the time series of the epidemic trajectory used in the calibration of the model. Here, *t*_*j*_ are the time points for the time series data, *n*_*d*_ is the number of observations, and each $y_{t_{j}},j=1,2,\ldots ,n_{d}$, corresponds to the time series data. Let *f*(*t*, Θ) denote the expected curve of the epidemic trajectory. We can estimate the set of model parameters, denoted by Θ, by fitting the model solution to the observed data via nonlinear least squares [11]; within MATLAB, set the parameter <method1> to 0 in the options_fit.m or options_forecast.m files. Least squares estimation is achieved by searching for the set of parameters $\hat{\Theta }$ that minimizes the sum of squared differences between the observed data $y_{t_{j}}=y_{t_{1}},y_{t_{2}}\ldots \ldots y_{t_{n_{d}}}$ and the best fit of the model (model mean) which corresponds to *f*(*t*, Θ). That is, Θ is estimated by $\hat{\Theta }=\text{arg min}\sum_{j=1}^{n_{d}}{\left(f\left(t_{j},\Theta \right)-y_{t_{j}}\right)}^{2}$.

This parameter estimation method weights each data point equally and does not require a specific distributional assumption for *y*_*t*_, except for the first moment *E*[*y*_*t*_]=*f*(*t*_*i*_; *Θ*). That is, the mean of the observed data at time *t* is equivalent to the expected count denoted by *f*(*t*, Θ) at time *t* [12]. This method yields asymptotically unbiased point estimates regardless of any misspecification of the variance-covariance error structure. Hence, the estimated model mean $f\left(t_{i},\hat{\Theta }\right)$ yields the best fit to the observed data $y_{t_{i}}$ in terms of the squared L2 norm. We can use the *fmincon* function in MATLAB to set the optimization problem. In addition to the underlying normal distribution assumption linked to nonlinear least squares fitting (method1=0), the uncertainty of the parameters can be quantified using a Poisson or negative binomial error structure using parameter <dist1> as described in the next section. Finally, we also employ MATLAB’s MultiStart feature to specify the number of random initial guesses of the model parameters using the parameter <numstartpoints> in the options_fit.m or options_forecast.m files to thoroughly search for the best-fit parameter estimates.

In addition to nonlinear least squares fitting, we can also estimate model parameters via maximum likelihood estimation (MLE) [13] with specific assumptions about the error structure in the data (e.g., Poisson, Negative Binomial) via parameter <method1>. The log-likelihood expressions derived for Poisson and negative binomial error structures are given below.

#### a) Poisson

For a Poisson error structure, the full log-likelihood of Poisson (i.e., method1=1) is given by:

$$\overset{n}{\underset{i=1}{\sum }}\left\{y_{i}ln\left(\mu_{i}\right)-ln\left(y_{i}!\right)-\mu_{i}\right\}$$

The number of parameters is equal to the number of parameters estimated for the sub-epidemic model based on ordinary differential equations.

#### b) Negative binomial

Let *r* > 0 denote the number of failures until the experiment is stopped and *p* ∈ [0, 1] denote the success probability in each experiment. Then the number of successes *y* before the *r*-th failure occurs has a **negative binomial** distribution:

$$f(y|r,p)=\left(\begin{array}{c} r+y-1\\ y \end{array}\right)p^{y}{\left(1-p\right)}^{r}=\frac{1}{y!}\overset{y-1}{\underset{j=0}{\prod }}(j+r).p^{y}{\left(1-p\right)}^{r}$$

with mean $\text{mean}\;=\mu =\frac{rp}{\left(1-p\right)}$, $\text{variance}=\sigma^{2}=\frac{rp}{{\left(1-p\right)}^{2}}>\mu$. For n observations *y*_1_, …, *y*_*n*_, the full log-likelihood is

(1.1) $$l(r,p)=\overset{n}{\underset{i=1}{\sum }}\left\{\left\{\overset{y_{i}-1}{\underset{j=0}{\sum }}ln(j+r)\right\}+y_{i}ln\left(p_{i}\right)+rln\left(1-p_{i}\right)-ln\left(y_{i}!\right)\right\}$$

If we want to express the distribution with *μ* and *σ*^2^, we can substitute $p=1-\frac{\mu }{\sigma^{2}}$ and $r=\frac{\mu^{2}}{\sigma^{2}-\mu }$.

There are different types of variances commonly used in a negative binomial distribution. If the variance scales linearly with the mean, then *σ*^2^ = *μ* + *αμ*, (i.e., method1=3 in options_fit.m or options_forecast.m), then $p=\frac{\alpha }{1+\alpha }$ and *r* = *μ/α*. The full log-likelihood (1.1) can be expressed as follows:

(1.2) $$l(\theta ,\alpha )=\overset{n}{\underset{i=1}{\sum }}\left\{\left\{\overset{y_{i}-1}{\underset{j=0}{\sum }}ln\left(j+\alpha^{-1}f\left(t_{i},\theta \right)\right)\right\}+y_{i}ln(\alpha )-\left(y_{i}+\alpha^{-1}f\left(t_{i},\theta \right)\right)ln(1+\alpha )-ln\left(y_{i}!\right)\right\}$$

If the variance scales quadratically with the mean, then *σ*^2^ = *μ* + *αμ*^2^ (ie., method1=4 in options_fit.m or options_forecast.m), then $p=\frac{\alpha \mu }{1+\alpha \mu }$ and *r* = 1/*α*.

Let *μ* = *f*(*t*, *θ*) be the mean curve to be estimated from the differential equation. The full log-likelihood (1.1) can be expressed as follows:

(1.3) $$l(\theta ,\alpha )=\sum_{i=1}^{n}\left\{\left\{\sum_{j=0}^{y_{i}-1}ln\left(j+\alpha^{-1}\right)\right\}+y_{i}ln\left(\alpha f\left(t_{i},\theta \right)\right)-\left(y_{i}+\alpha^{-1}\right)ln\left(1+\alpha f\left(t_{i},\theta \right)\right)-ln\left(y_{i}!\right)\right\}$$

The more general form of variance is *σ*^2^ = *μ* + *αμ*^*d*^ (i.e., method1=5 in options_fit.m or options_forecast.m) with any −∞ < *d* < ∞. Then the full log-likelihood (1.1) can be expressed as follows:

(1.4) $$l(\theta ,\alpha )=\overset{n}{\underset{i=1}{\sum }}\left[\left\{\overset{y_{i}-1}{\underset{j=0}{\sum }}ln\left(j+\alpha^{-1}\mu_{i}^{\;2-d}\right)\right\}+y_{i}ln\left(\alpha \mu_{i}^{\;d-1}\right)-\left(y_{i}+\alpha^{-1}\mu_{i}^{\;2-d}\right)ln\left(1+\alpha \mu_{i}^{\;d-1}\right)-ln\left(y_{i}!\right)\right]$$

where *μ*_*i*_ = *f*(*ti*, *θ*)

The number of parameters is 1 plus the number of parameters in the dynamical model based on ordinary differential equations (ODE) for (1.2) ~ (1.3), and 2 plus the number of parameters in the dynamical model for (1.4) if d is also estimated via MLE.

##### Parametric bootstrapping

To quantify parameter uncertainty, we follow a parametric bootstrapping approach which allows the computation of standard errors and related statistics without closed-form formulas [14]. We generate *B* bootstrap samples from the best-fit model $f(t,\hat{\Theta })$, with an assumed error structure, specified using parameter <dist1> in the options_fit.m or options_forecast.m files, to quantify the uncertainty of the parameter estimates and construct confidence intervals. Typically, the error structure in the data is modeled using a probability model such as the Poisson or negative binomial distribution. Using nonlinear least squares (method1=0), in addition to a normally distributed error structure (dist1=0), we can also assume a Poisson (dist1=1) or a negative binomial distribution (dist1=2), whereby the variance-to-mean ratio is empirically estimated from the time series. To estimate this constant ratio, we group a fixed number of observations (e.g., 7 observations for daily data into a bin across time), calculate the mean and variance for each bin, and then estimate a constant variance-to-mean ratio by calculating the average of the variance-to-mean ratios over these bins. Using maximum likelihood estimation, we can estimate parameter uncertainty for Poisson and negative binomial error structures in the data (methodl1=1 & dist1=1 for Poisson and method1=3 & dist1=3, method1=4 & dist1=4, and method1=5 & dist1=5 for the different negative binomial error structures described above).

Using the best-fit model $f(t,\hat{\Theta })$, we generate *B*-times replicated simulated datasets of size *n*_*d*_, where the observation at time *t*_*j*_ is sampled from the corresponding distribution specified by <dist1>. Next, we refit the model to each of the *B* simulated datasets to re-estimate the parameters for each dataset. The new parameter estimates for each realization are denoted by ${\hat{\Theta }}_{b}$, where *b* = 1, 2, …, *B*. Using the sets of re-estimated parameters (${\hat{\Theta }}_{b}$), it is possible to characterize the empirical distribution of each parameter estimate, calculate the variance, and construct confidence intervals for each parameter. The resulting uncertainty around the model fit can similarly be obtained from $f\left(t,{\hat{\Theta }}_{1}\right)$, $f\left(t,{\hat{\Theta }}_{2}\right)$, …, $f\left(t,{\hat{\Theta }}_{B}\right)$. We characterize the uncertainty using 300 bootstrap realizations (i.e., parameter B=300 in the options_fit.m or options_forecast.m files).

##### Model-based forecasts with quantified uncertainty

Based on the best-fit model $f(t,\hat{\Theta })$, we can make days *h* ahead forecasting using $f(t+h,\hat{\Theta })$. The uncertainty of the forecasted value can be obtained using the previously described parametric bootstrap method. Let

$$f\left(t+h,{\hat{\Theta }}_{1}\right),f\left(t+h,{\hat{\Theta }}_{2}\right),\ldots ,f\left(t+h,{\hat{\Theta }}_{B}\right)$$

denote the forecasted value of the current state of the system propagated by a horizon of *h* time units, where ${\hat{\Theta }}_{b}$ denotes the estimation of parameter set Θ from the *b*_*th*_ bootstrap sample. We can use these values to calculate the bootstrap variance to measure the uncertainty of the forecasts and use the 2.5% and 97.5% percentiles to construct the 95% prediction intervals (PI). We can set the forecasting horizon (*h*) using the parameter <forecastingperiod1> in the options_forecast.m file.

For illustration, we fit the models through the nonlinear least squares method with a normal error structure (i.e., method1=0 and dist1=0) for the monkeypox data. In the options_fit.m or options_forecast.m files, the values of the parameters related to the parameter estimation method and parametric bootstrapping follow:


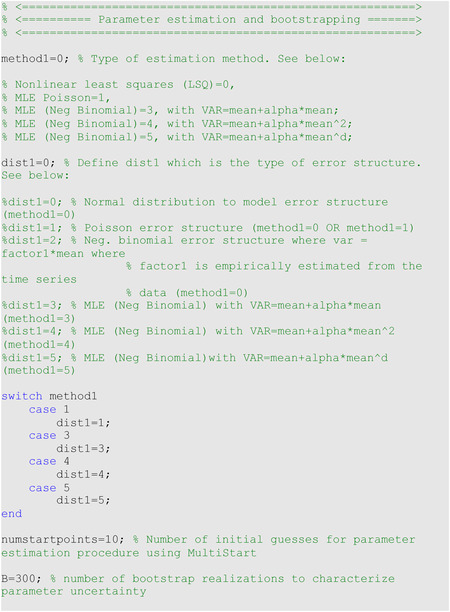


### Phenomenological growth models

Below we describe the growth models included in the toolbox where *C*(*t*) denotes the cumulative case count at time *t*.

### Generalized-growth model (GGM)

Models commonly used to study the growth pattern of infectious disease outbreaks often assume exponential growth in the absence of control interventions (compartmental models, for example); however, growth patterns are likely slower than exponential for some diseases depending on the transmission mode and population structure. For example, Ebola spreads via close contact so sub-exponential growth patterns would be expected in a constrained population contact structure [15]. The generalized growth model (GGM) [6] includes a “deceleration of growth” parameter, p (range: [0, 1]), that relaxes the assumption of exponential growth. A value of p=0 represents constant (linear) growth, while a value of p=1 indicates exponential growth. If 0<p<1, the growth pattern is characterized as sub-exponential or polynomial.

The GGM is as follows:

$$\frac{dC(t)}{dt}=\dot{C}(t)=rC{\left(t\right)}^{p}$$

where C(t) describes the cumulative number of cases at time t, $\dot{C}(t)$ is the incidence curve, r is the growth rate parameter (r>0), and p, again, is the deceleration of growth parameter [6]. For this model, we estimate Θ = (*r*, *p*). This model can be selected by setting flag1=0 in the options_fit.m and options_forecast.m files.

### Generalized Logistic growth model (GLM)

The Generalized Logistic growth model (GLM) has three parameters and is given by:

$$\frac{dC(t)}{dt}=C^{\prime }(t)=rC^{p}(t)\left(1-\frac{C(t)}{K_{0}}\right)$$

The growth scaling parameter, *p*, is also used in the GLM to model a range of early epidemic growth profiles ranging from constant incidence (*p* = 0), polynomial (0 < *p* < 1), and exponential growth dynamics (*p* = 1). When *p* = 1, this model reduces to the logistic growth model (flag1=3). The remaining model parameters are as follows: *r* is the growth rate, and *K*_0_ is the final cumulative epidemic size. For this model, we estimate Θ=(*r*, *p*, *K*_0_) where *f*(*t*, Θ) = *C*′(*t*), and the initial number of cases *C*(0) is fixed according to the first observation in the data. The GLM model has been employed to generate short-term forecasts of Zika, Ebola, and COVID-19 epidemics [2, 4, 5, 16]. This model can be selected by setting flag1=1 in the options_fit.m and options_forecast.m files.

### Richards model (RIC)

The well-known Richards model is an extension of the simple logistic growth model and relies on three parameters. It extends the simple logistic growth model by incorporating a scaling parameter, *α*, that measures the deviation from the symmetric simple logistic growth curve [1, 17, 18]. The Richards model is given by the differential equation:

$$\frac{dC(t)}{dt}=rC(t)\left[1-{\left(\frac{C(t)}{K_{0}}\right)}^{a}\right]$$

where *r* is the growth rate, *α* is a scaling parameter and *K*_0_ is the final epidemic size. The Richards model has been employed to generate short-term forecasts of SARS, Zika, Ebola, and COVID-19 epidemics [2, 4, 5, 16, 19]. For this model, we estimate Θ = (*r*, *α*, *K*_0_). This model can be selected by setting flag1=4 in the options_fit.m and options_forecast.m files. A 4-parameter extension of the Richards model is the generalized Richards model (flag1=2), which incorporates the growth scaling parameter *p* used in the generalized-logistic growth model.

#### Quality of model fit

To assess the quality of model fit, we can compare the *AIC*_*c*_ (Akaike Information Criterion) values of the best-fit models. The *AIC*_*c*_ is given by [20, 21]:

$$AIC_{c}=-2log(\text{likelihood})+2m+\frac{2m(m+1)}{n_{d}-m-1}$$

where *m* is the number of model parameters and *n*_*d*_ is the number of data points. Specifically for normal distribution, the *AIC*_*c*_ is

$$AIC_{c}=n_{d}log(SSE)+2m+\frac{2m(m+1)}{n_{d}-m-1}$$

where $SSE=\sum_{j=1}^{n_{d}}{\left(f\left(t_{j},\hat{\Theta }\right)-y_{t_{j}}\right)}^{2}$. Thus, this metric is used for model selection and accounts for model complexity in terms of the number of model parameters. In the options_fit.m and options_forecast.m files, the values of the parameters related to the selection of the growth model follow:


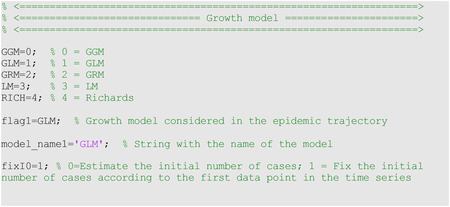


In the next section, we describe four comprehensive performance metrics that can be used to assess both calibration and forecasting performance. Specifically, the mean absolute error (MAE) and the mean squared error (MSE) are used to assess the performance of point forecasts while the coverage of the 95% prediction interval and the weighted interval score (WIS) evaluate the performance of distributional forecasts by accounting for uncertainty in model fit and predictions.

### Performance metrics

To assess calibration and forecasting performance, we used four performance metrics: the mean absolute error (MAE), the mean squared error (MSE), the coverage of the 95% prediction intervals, and the weighted interval score (WIS) [22]. In the options_forecast.m file, the parameter <getperformance> is a boolean variable (0/1) to indicate whether the user wishes to compute the performance metrics of the forecasts.

The *mean absolute error* (MAE) is given by:

$$\text{MAE}=\frac{1}{N}\overset{N}{\underset{h=1}{\sum }}\left|f\left(t_{h},\hat{\Theta }\right)-y_{t_{h}}\right|$$

where *t*_*h*_ are the time points of the time series data [23], and N is the length of the calibration period or forecasting period. Similarly, the *mean squared error* (MSE) is given by:

$$\text{MSE}=\frac{1}{N}\overset{N}{\underset{h=1}{\sum }}{\left(f\left(t_{h},\hat{\Theta }\right)-y_{t_{h}}\right)}^{2}.$$

The coverage of the *95% prediction interval* (*PI*) corresponds to the fraction of data points that fall within the 95% PI, calculated as

$$95\%\text{ PI coverage}=\frac{1}{N}\sum_{t=1}^{n}1\left\{Y_{t}>L_{t}\cap Y_{t}<U_{t}\right\}$$

where *L*_*t*_ and *U*_*t*_ are the lower and upper bounds of the 95% PIs, respectively, *Y*_*t*_ are the data and **1** is an indicator variable that equals 1 if *Y*_*t*_ is in the specified interval and 0 otherwise.

The *weighted interval score* (WIS) [22, 24], which is a proper score recently embraced for quantifying model forecasting performance in epidemic forecasting studies [25–29], provides quantiles of predictive forecast distribution by combining a set of Interval Score (IS) for probabilistic forecasts. An IS is a simple proper score that requires only a central (1−*α*)×100% PI [30] and is described as

$$IS_{\alpha }(F,y)=(u-l)+\frac{2}{\alpha }\times (l-y)\times 1(y<l)+\frac{2}{\alpha }\times (y-u)\times 1(y>u).$$

In this equation **1** refers to the indicator function, meaning that **1**(*y* < *l*) = 1 if *y* < *l* and 0 otherwise. The terms *l* and *u* represent the $\frac{\alpha }{2}$ and $\;1-\frac{\alpha }{2}$ quantiles of the forecast *F*. The IS consists of three distinct quantities:

1. The sharpness of *F*, given by the width *u* − *l* of the central (1 − *α*) × 100% PI.
2. A penalty term $\frac{2}{\alpha }\times (l-y)\times 1(y<l)$ for the observations that fall below the lower end point *l* of the (1 − *α*) × 100% PI. This penalty term is directly proportional to the distance between *y* and the lower end *l* of the PI. The strength of the penalty depends on the level *α*.
3. An analogous penalty term $\frac{2}{\alpha }\times (y-u)\times 1(y>u)$ for the observations falling above the upper limit *u* of the PI.

To provide more detailed and accurate information on the entire predictive distribution, we report several central PIs at different levels (1 − *α*_1_) < (1 − *α*_2_) < ⋯ < (1 − *α*_*K*_) along with the predictive median, $\tilde{y}$, which can be seen as a central prediction interval at level 1 − *α*_0_ → 0. This is referred to as the WIS, and it can be evaluated as follows:

$$WIS_{\alpha_{0:K}}(F,y)=\frac{1}{K+\frac{1}{2}}.\left(w_{0}.\left|y-\tilde{y}\right|+\overset{K}{\underset{k=1}{\sum }}w_{k}.IS_{\alpha_{k}}(F,y)\right)$$

where, $w_{k}=\frac{\alpha_{k}}{2}$ for *k* = 1, 2, …. *K* and $w_{0}=\frac{1}{2}$. Hence, WIS can be interpreted as a measure of how close the entire distribution is to the observation in units on the scale of the observed data [31, 32].

In the options_forecast.m file, we can specify the parameters related to the epidemic forecasts including the forecasting horizon and an indicator variable to specify whether the forecasting performance metrics should be computed:


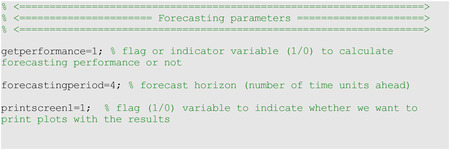


### Rolling window analysis

A rolling window analysis can be useful to assess the stability of the model parameters and forecasts over time and requires the specification of three parameters in the options_fit.m or options_forecast.m files: The start time (<tstart1>) of the first rolling window, the window size (<windowsize1>), and the end time (<tend1>) which corresponds to the start time of the last rolling window. Hence, the first rolling window contains observations for period <start1> to <tstart1> + <windowsize1> −1, the second rolling window contains observations for period <start1> + 1 through <windowsize1>, and so on. Therefore, <windowsize1> corresponds to the length of the calibration period for each model fit. The outputs obtained from the rolling window analysis correspond to the parameter estimates and their uncertainty for each rolling window subsample. A plot of the parameter estimates over the rolling windows can help examine how the estimates change with time. The parameters can be specified in the options_fit.m and options_forecast.m files as shown below, but they can also be passed as input parameters to the fitting and forecasting functions as described in the following.


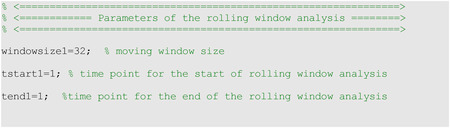


## Results and Discussion

### Fitting the models to data with quantified uncertainty

The function Run_Fit_GrowthModels.m can be used to fit one of the phenomenological growth models to data with quantified uncertainty. The function uses the input parameters provided by the user in the options_fit.m file. However, the function may receive the parameters related to the rolling window analysis (<tstart1>, <tend1>, and <windowsize1>) as passing input parameters with the remaining inputs accessed from the options_fit.m file.

For example, we can fit the generalized logistic growth model (flag1=1 in options_fit.m file) to the weekly incidence curve of monkeypox in the USA located in the input folder (data file path: ./input/ Most_Recent_Timeseries_US-CDC.txt). To that end, we assume a normal error structure (i.e., dist1=0 in the options_fit.m file) and examine the fit of the model to the data. Since the monkeypox epidemic curve comprises 32 weeks of data, we can pass the rolling window parameters to the function call in MATLAB as follows:


>> **Run_Fit_subepidemicFramework**(1, 1, 32) 


**Figure 1.**
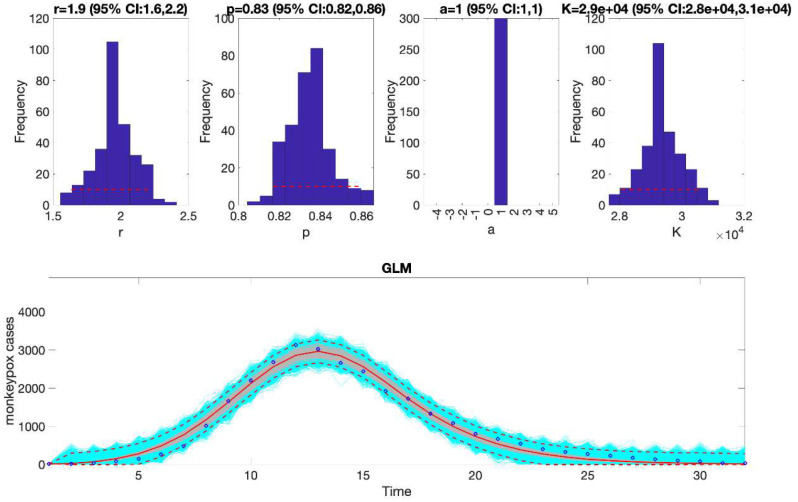
Fitting the generalized logistic-growth model to the entire incidence curve of the monkeypox epidemic in the USA, week of 12 May 2022 through the week of 15 December 2022. The model provides a good fit to the entire incidence curve. The model supports sub-exponential growth dynamics (i.e., p ~ 0.8–0.9) and the epidemic size parameter was estimated at ~ 28,000–31,000 cases. The solid red line is the median model fit. The red lines correspond to the mean of the model fits obtained from the parametric bootstrapping with 300 bootstrap realizations whereas the cyan lines indicate the predictive uncertainty around the model fit, and the dashed lines correspond to the 95% prediction intervals.

In the above call to the function, tstart1=1, tend1=1, and windowsize=32. Hence, this function will generate a single model fit and store several output MATLAB files related to the model fit, parameter estimates, and the quality of model fit in the output folder. For each model fit, it will also generate a figure (Figure 1) with the model fit and the corresponding empirical distributions of the parameters.

### Plot the mean model fits and compute calibration performance metrics

Once the Run_Fit_subepidemicFramework.m has been executed, the user can run the function plotFit_GrowthModels.m to display the model fit and the empirical distribution of the parameters. It also saves output .csv files in the output folder with the model fit, the parameter estimates including 95% CIs, and the calibration performance metrics. The call to the function from MATLAB’s command line follows:


>> **plotFit_GrowthModels**(1, 1, 32) 


This function will store the following .csv files in the output folder:

1. The model fit to the data:

Fit-flag1-1-i-1-fixI0-1-method-0-dist-0-calibrationperiod-32-horizon-0-monkeypox-cases.csv
2. Model parameter estimates:

parameters-rollingwindow-flag1-1-fixI0-1-method-0-dist-0-tstart-1-tend-1-calibrationperiod-32-horizon-0-monkeypox-cases.csv
3. Calibration performance metrics:

parameters-rollingwindow-flag1-1-fixI0-1-method-0-dist-0-tstart-1-tend-1-calibrationperiod-32-horizon-0-monkeypox-cases.csv

**Figure 2.**
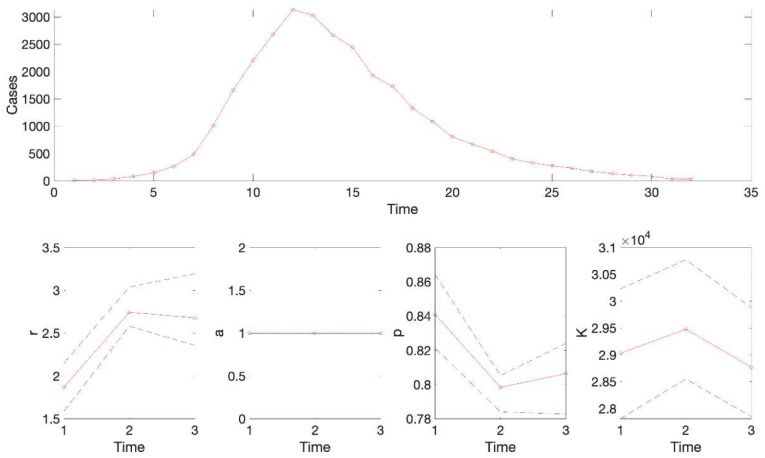
Results of the rolling window analysis after running the function plotFit_GrowthModels(1, 3, 30). The top panel shows the monkeypox epidemic curve in the US for reference. The bottom panels show the temporal sequence of parameter estimates (-o-, red line), and their 95% CIs (blue dashed lines) for three different moving time windows (1–30, 2–31, 3–32).

For this example, the resulting calibration performance metrics indicate that the 95% prediction intervals include all the data points comprising the epidemic curve of monkeypox in the USA (i.e., coverage of the 95% PI is 100%) (see Table 2). This function also plots the temporal sequence of parameter estimates and their uncertainty obtained from the rolling-window analysis whenever the value of the parameter <tend1> is greater than parameter <tstart1>. For instance, after running the function Run_Fit_GrowthModels(1, 3, 30) to generate a rolling window analysis of model fits with window size 30 and start times at 1, 2, and 3, we can run the function plotFit_GrowthModels(1, 3, 30) from MATLAB’s command line to generate the rolling window analysis plot (Figure 2).

**Figure 3.**
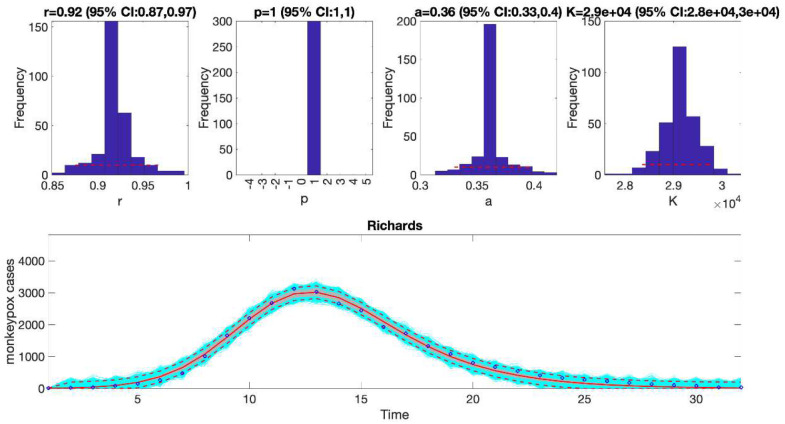
The fit of the Richards model to the entire incidence curve of the monkeypox epidemic in the USA, the week of 12 May 2022 through the week of 15 December 2022. The model provides a good fit to the entire incidence curve. The epidemic size parameter was estimated at ~ 28,000–30,000 cases, similar to that estimated using the generalized logistic-growth model. In the lower panel, blue dots are the data points whereas the solid red line is the median model fit. The red lines correspond to the mean of the model fits obtained from the parametric bootstrapping with 300 bootstrap realizations whereas the cyan lines indicate the predictive uncertainty around the model fit, and the dashed lines correspond to the 95% prediction intervals.

We can also assess the fit of the Richards model to the monkeypox incidence curve (flag1=4 in options_fit.m file) and compare the quality of the model fit to that obtained using the generalized logistic growth model using the performance metrics. Figure 3 shows the fit of the Richards model to the epidemic curve and the empirical distribution of the parameters.

The calibration performance metrics of the generalized logistic growth model and the Richards model (see Table 2) indicate that the Richards model yields a better fit to the data in terms of the MAE, MSE, and WIS while both models achieved a 100% coverage of the 95% prediction interval.

### Plotting and assessing model-based forecasts

To generate a forecast, we can use the function Run_Forecasting_GrowthModels.m. This function uses the input parameters provided by the user in the options_forecast.m file. However, the function can also receive <tstart1>, <tend1>, <windowsize1>, and <forecastingperiod> as passing input parameters with the remaining input parameters accessed from the options_forecast.m file.

For example, we can fit the generalized logistic growth model (flag1=1 in options_forecast.m file) to the first 10 weeks of the monkeypox epidemic in the USA assuming a normal error structure (i.e, dist1=0 in options_forecast.m file) and generate a 4-week ahead prediction by running the function from MATLAB’s command line as follows:


>> **Run_Forecasting_GrowthModels**(1, 1, 10, 4) 


**Figure 4.**
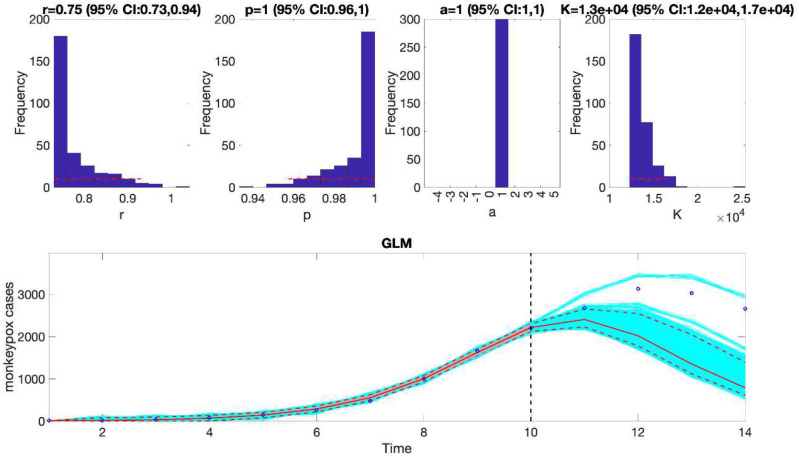
The generalized logistic-growth model fit and 4-week ahead forecast based on the first 10 weeks of the monkeypox epidemic in the USA, the week of 12 May 2022 through the week of 14 July 2022. The model fit is consistent with early exponential growth dynamics (i.e., p~1) and predicts an epidemic size at K ~ 120,000 to 170,000 cases. The solid red line is the median model fit. The blue dots indicate the data points. The red lines correspond to the mean of the model fits obtained from the parametric bootstrapping with 300 bootstrap realizations whereas the cyan lines indicate the predictive uncertainty around the model fit, and the dashed lines correspond to the 95% prediction intervals. The vertical dashed line separates the 10-week calibration (left) and the 4-week ahead forecasting period (right). Overall, the 4-week ahead forecast underpredicted the trajectory of the epidemic.

**Figure 5.**
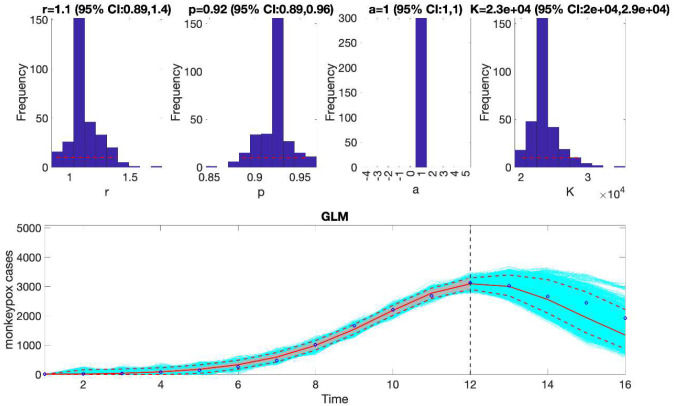
The generalized logistic-growth model fit and 4-week ahead forecast based on the first 12 weeks of the monkeypox epidemic in the USA, the week of 12 May 2022 through the week of 28 July 2022. The model fit is consistent with early sub-exponential growth dynamics (i.e., p~0.92) and predicts an epidemic size at K ~ 20,000 to 29,000 cases. The solid red line is the median model fit. The blue dots indicate the data points. The red lines correspond to the mean of the model fits obtained from the parametric bootstrapping with 300 bootstrap realizations whereas the cyan lines indicate the predictive uncertainty around the model fit, and the dashed lines correspond to the 95% prediction intervals. The vertical dashed line separates the 12-week calibration (left) and the 4-week ahead forecasting period (right). Overall, the 4-week ahead forecast using the GLM tracked well the trajectory of the epidemic.

This function will generate a single model fit and store several output MATLAB files related to the model fit and forecast, parameter estimates, and the calibration and forecasting performance metrics. It will also generate a figure (Figure 4) with the model fit and 4-week ahead forecast and the corresponding empirical distributions of the parameters. Overall, the 4-week ahead forecast shown in Figure 4 underpredicted the trajectory of the epidemic. For comparison, Figure 5 also shows the model fit and 4-week ahead forecast when the model is calibrated using the first 12 weeks of the epidemic curve instead of the first 10 weeks. The 4-week ahead forecast based on the first 12 weeks of data clearly forecasted better the epidemic curve than the one based on the first 10 weeks of data.

Once the user has executed the function Run_Forecasting_GrowthModels, the function plotForecast_GrowthModels can be used to plot the model-based forecast and the performance metrics of the forecast (MSE, MAE, 95% PI, WIS) based on the inputs indicated in the options_forecast.m file. However, this function can also receive <tstart1>, <tend1>, <windowsize1>, and <forecastingperiod> as passing input parameters while the remaining inputs are retrieved from the options_forecast.m file. Moreover, the data associated with the forecasts, the parameter estimates, as well as the calibration and forecasting performance metrics, are saved as .csv files in the output folder. For example, the following line illustrates the execution of the function from MATLAB’s command window:


>> **plotForecast_GrowthModels**(1, 1, 10, 4) 


**Figure 6.**
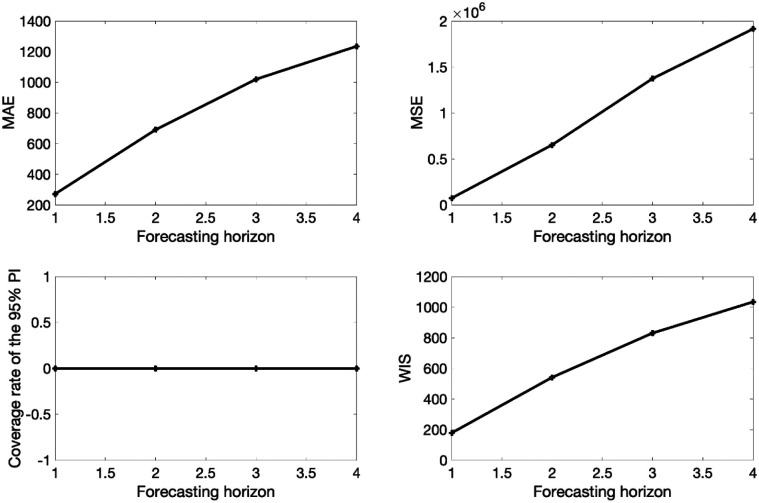
Forecasting performance metrics associated with the 4-week ahead forecasts obtained from fitting the generalized logistic growth model to the first 10 weeks of the monkeypox epidemic in the USA.

This function plots the model fit based on a 10-week calibration period and a 4-week ahead forecast as well as the empirical distribution of the estimated parameters (Figure 4). It also displays the associated forecasting performance metrics (see Figure 6).

**Figure 7.**
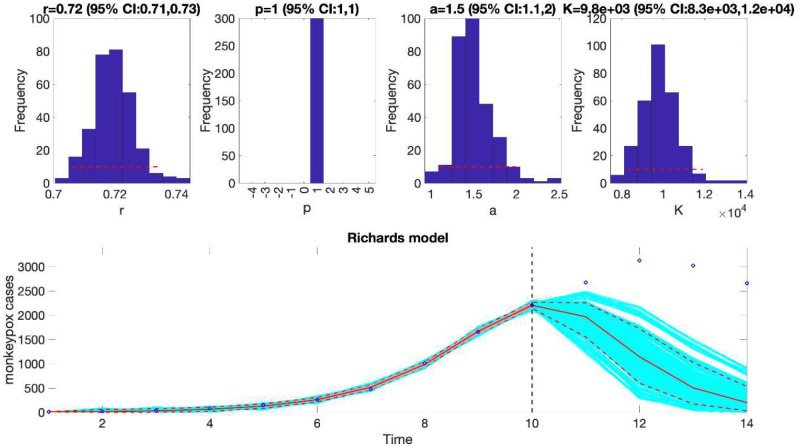
The Richards model fit, and 4-week ahead forecast based on the first 10 weeks of the monkeypox epidemic in the USA, the week of 12 May 2022 through the week of 14 July 2022. The model fit estimates the epidemic size at K ~ 8,300 to 12,000 cases. The solid red line is the median model fit. The blue dots indicate the data points. The red lines correspond to the mean of the model fits obtained from the parametric bootstrapping with 300 bootstrap realizations whereas the cyan lines indicate the predictive uncertainty around the model fit, and the dashed lines correspond to the 95% prediction intervals. The vertical dashed line separates the 10-week calibration period (left) and the 4-week ahead forecast period (right). Overall, the 4-week ahead forecast underpredicted the trajectory of the epidemic.

Similarly, we can also generate the forecast using the Richards model by specifying flag1=4 in the options_forecast.m file and compare the forecasting performance of this model with that obtained using the generalized logistic growth model using the performance metrics. Figure 7 shows the forecast of the Richards model to the first 10 weeks of the epidemic curve and the empirical distribution of the parameters.

The forecasting performance metrics of the generalized logistic growth model and the Richards model based on the first 10 weeks of the epidemic curve (shown in Table 3) indicate that the generalized logistic growth model outperformed the Richards model in terms of forecasting performance.

## Conclusion

In this tutorial-based primer, we have introduced a MATLAB toolbox to fit and forecast time-series trajectories using phenomenological dynamic growth models with applications in natural and social sciences. In particular, the models included in the toolbox have been frequently applied to characterize and forecast epidemic trajectories in near real-time [2, 4–6, 8, 16, 19]. The toolbox can be used as part of the curriculum of student training in mathematical biology, applied statistics, infectious disease modeling, and specialty courses in epidemic modeling and time-series forecasting.

## Availability and requirements

Project name: Forecasting time series with phenomenological growth models

Project home page: https://github.com/gchowell/forecasting_growthmodels

Operating system(s): Platform independent

Programming language: MATLAB

Other requirements: NA

License: This program is free software: it can be redistributed or modified under the GNU Public

License as published by the Free Software Foundation, version 3 of the License

Any restrictions to use by non-academics: None

## Figures and Tables

**Table 1.** Description of the functions associated with the toolbox.

Function	Type	Role
Options_fit.m	User	Specifies the parameters related to model fitting including the characteristics of the time series data, the model, parameter estimation method, error structure, and calibration period
Options_forecast.m	User	Specifies the parameters related to model forecasting including the forecasting period, the calibration period, the characteristics of the time series data, the model, parameter estimation method, and error structure.
Run_Fit_GrowthModels.m	User	Fits a model to data with quantified uncertainty
Run_Forecasting_GrowthModels.m	User	Fits a model to data with quantified uncertainty and generates a model-based forecast with quantified uncertainty
plotFit_GrowthModels	User	Display the model fit and the empirical distribution of the parameters. It also saves output .csv files in the output folder with the model fit, the parameter estimates including 95% CIs, and the calibration performance metrics.
plotForecast_GrowthModels	User	Display the model-based forecast and the performance metrics of the forecast. Moreover, the data associated with the forecasts, the parameter estimates, as well as the calibration and forecasting performance metrics, are saved as .csv files in the output folder.
fit_model.m	Internal	Fit a model to the time-series data
getAICc.m	Internal	Computes the AIC_c_ for the model’s fit.
plotModifiedLogisticGrowthMethods1.m	Internal	Objective function definition for fitting a model to data
initialParams.m	Internal	Sets the initial guesses of the parameter values prior to model fitting
modifiedLogisticGrowth.m	Internal	Defines the models for numerical solution
AddErrorStructure.m	Internal	Generates realizations of the model fit with the specified error structure via parametric bootstrapping.
Computeforecastperformance.m	Internal	Computes performance metrics for the calibration and forecasting periods (MAE, MSE, coverage of the 95%PI)
computeWIS.m	Internal	Computes weighted interval score (WIS) to assess model performance during the calibration and forecasting periods.
getMeanVarianceRatio.m	Internal	Computes the average of the variance-to-mean ratios from the time series data to empirically characterize overdispersion using a negative binomial distribution when method1=0 and dist1=2.
computeQuantiles.m	Internal	Computes the quantiles from the uncertainty of the model separately for the calibration and forecasting periods.

**Table 2.** Calibration performance metrics quantifying how well the fits of the generalized logistic growth model and the Richards model captured the epidemic curve of monkeypox in the USA. The metrics indicate that the Richards model yields a better fit to the data in terms of the MAE, MSE, and WIS while both models achieved a 100% coverage of the 95% prediction interval.

Model	Calibration period	MAE	MSE	Coverage 95% PI	WIS
**Generalized logistic growth model**	32	109.64	17262.50	100.00	63.36
**Richards model**	32	70.84	7928.06	100.00	43.37

**Table 3.** Calibration and forecasting performance metrics obtained from the fits of the generalized logistic growth model and the Richards model based on the first 10 weeks of the monkeypox epidemic in the USA. The metrics indicate that the Richards model yields a better fit to the data (better calibration performance) than the generalized logistic growth model in terms of the MAE, MSE, and WIS. However, the generalized logistic growth model outperformed the Richards model in terms of forecasting performance.

Calibration performance					
Model	Calibration period	MAE	MSE	Coverage 95% PI	WIS
**Generalized logistic growth model**	10	17.67	800.70	90.00	13.20
**Richards**	10	11.90	301.50	100.00	8.55
Forecast performance					
Model	Forecasting period	MAE	MSE	Coverage 95% PI	WIS
**Generalized logistic growth model**	4	1234.70	1913137.40	0.00	1034.10
**Richards**	4	1919.63	4216607.63	0.00	1756.15

## Data Availability

The datasets analyzed during the current study include the U.S. Monkeypox Case Trends Reported to CDC data [https://www.cdc.gov/poxvirus/monkeypox/response/2022/mpx-trends.html]. The tutorial video is available in the Dropbox link https://www.dropbox.com/sh/esw2k1crt2exsfq/AABivzvuhov_dklOH6KmLh40a?dl=0.

## List of abbreviations

AIC: Akaike Information Criterion
ARIMA: Auto Regressive Integrated Moving Average
CDC: Centers for Disease Control and Prevention
CI: Confidence Interval
GGM: Generalized growth Model
GLM: Generalized Logistic growth Model
IS: Interval Score
MAE: Mean Absolute Error
MLE: Maximum Likelihood
MSE: Mean Squared Error
ODE: Ordinary Differential Equations
PI: Prediction Interval
RIC: Richards Model
SARS: Severe Acute Respiratory Syndrome
USA: United States of America
WIS: Weighted Interval Score
